# Supplementary figures and images for: Inhibition of type I PRMTs reforms muscle stem cell identity enhancing their therapeutic capacity
Source: eLife. 2023 Jun 7;12:RP84570. doi: 10.7554/eLife.84570 (PMC10328524; doi:10.7554/eLife.84570)

MS623

|             |   |   |   |   |   |   |   |   |   |    |
|-------------|---|---|---|---|---|---|---|---|---|----|
| DMSO        | 1 | 2 | 3 | 4 | 5 | 6 | 7 | 8 | 9 | 10 |
| 3 $\mu$ M   |   |   |   |   |   |   |   |   |   |    |
| 2.5 $\mu$ M |   |   |   |   |   |   |   |   |   |    |
| 2 $\mu$ M   |   |   |   |   |   |   |   |   |   |    |
| 1 $\mu$ M   |   |   |   |   |   |   |   |   |   |    |
| DMSO        |   |   |   |   |   |   |   |   |   |    |
| mem         |   |   |   |   |   |   |   |   |   |    |

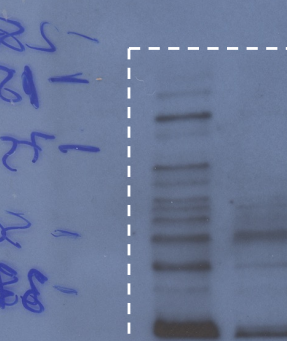

1  
2  
3  
4  
5

6  
7  
8  
9  
10

Supplement: Figure 1—figure supplement 1—source data 1. [file elife-84570-fig1-figsupp1-data1.zip › Figure 1-Source data.pdf]

Figure 6- Source data

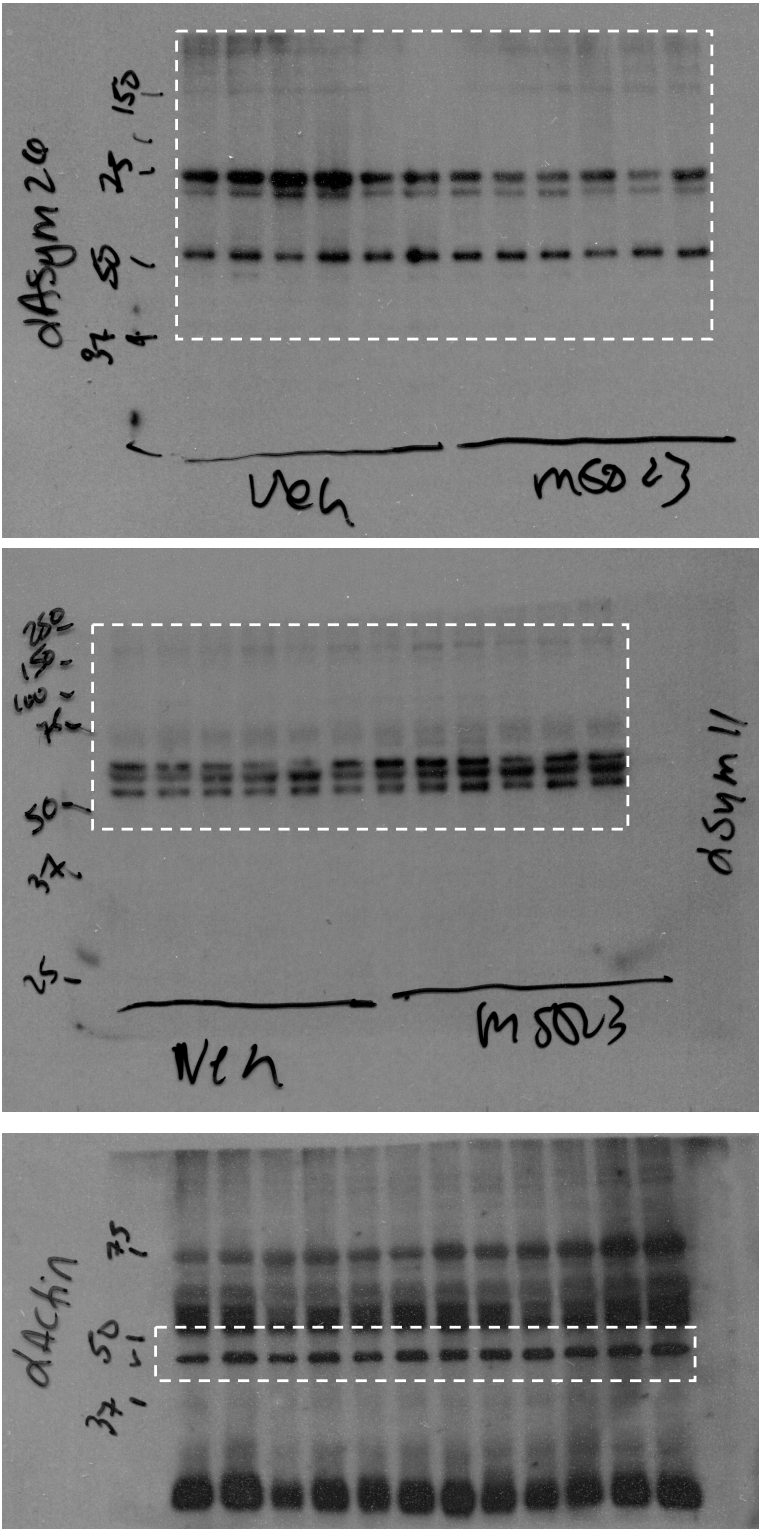

Supplement: Figure 6—figure supplement 1—source data 1. [file elife-84570-fig6-figsupp1-data1.zip › Figure6-SourceData.pdf]
